# Synthesis of Fe_3_O_4_/PDA Nanocomposites for Osteosarcoma Magnetic Resonance Imaging and Photothermal Therapy

**DOI:** 10.3389/fbioe.2022.844540

**Published:** 2022-03-09

**Authors:** Yifei Zhang, Rende Ning, Wei Wang, Yejin Zhou, Yao Chen

**Affiliations:** ^1^ Department of Human Anatomy, West China School of Basic Medicine & Forensic Medicine, Sichuan University, Chengdu, China; ^2^ Department of Orthopaedics, The Third Affiliated Hospital of Anhui Medical University, Hefei, China

**Keywords:** nanocomposites, osteosarcoma, metastasis, photothermal therapy, diagnosis

## Abstract

Osteosarcomas commonly develop in the metaphysis of the long diaphysis, resulting in pronounced malignancy and high rates of early pulmonary metastasis. At present, osteosarcoma patients exhibit relatively poor survival rates owing these metastases and to the emergence of tumor chemoresistance. As such, there is an urgent need to identify other approaches to treating affected patients. Herein, we synthesized Fe_3_O_4_@PDA nanocomposites that exhibited excellent biocompatibility and low toxicity in human and animal model systems. The resultant nanoparticles were able to improve T2 magnetic resonance imaging and to enhance the signal-to-noise ratio associated with osteosarcoma tumors in animal models. Moreover, we were able to successfully leverage these Fe_3_O_4_@PDA particles as a photothermal agent capable of significantly inhibiting the growth of tumors and preventing their metastasis to the lung compartment. Together, these results highlight a novel therapeutic platform that has the potential to guide both the more effective diagnosis and treatment of osteosarcoma patients in clinical applications.

## Introduction

Osteosarcoma (OS) is the most prevalent form of primary bone malignancy among children and young adults (Kansara et al., 2014; Isakoff et al., 2015; Roessner et al., 2021). These tumors often develop in the long diaphysis, with tumors of the proximal tibia and distal femur being particularly common (Bielack et al., 2002; Whelan et al., 2012; Kollar et al., 2019). OS is associated with a highly aggressive and malignant disease course characterized by high rates of pulmonary metastasis, with ∼80% of metastatic nodules ultimately developing in the lungs (Bielack et al., 2002; PosthumaDeBoer et al., 2011). The early diagnosis of osteosarcoma is difficult due to the limitations of available imaging technologies and the atypical symptoms associated with early-stage disease. Even with a combination of surgery and adjuvant chemotherapy, only 65–70% of OS patients achieve curative outcomes (PosthumaDeBoer et al., 2011; Whelan and Davis, 2018), and 5-year overall survival rates for metastatic OS patients are just 20% (Meyers et al., 2011; Doyle, 2014; Setsu, 2015). These low survival rates are primarily attributable to a combination of high rates of pulmonary metastasis and the frequent emergence of chemoresistance in treated patients (Snyder et al., 1990; Gadd et al., 1993; Kempf-Bielack et al., 2005; Simon et al., 2005). Over the past four decades, little progress has been made in improving OS patient survival rates (Gill and Gorlick, 2021), underscoring the need for the development of novel treatment approaches for affected patients.

Photothermal therapy (PTT) is a noninvasive therapeutic modality in which the energy-absorbing properties of particular agents, known as photosensitizers, are leveraged such that when they are exposed to near-infrared (NIR) light, they convert that NIR energy into heat to selectively ablate tumor cells (Wang et al., 2015; Wang et al., 2016; Zhang J. et al., 2019; Shramova et al., 2020; Bu et al., 2021; Gao et al., 2021; Zhang et al., 2021). Owing to its promise, NIR laser-induced PTT has emerged as a prominent form of noninvasive tumor treatment (Hou et al., 2018; Liu et al., 2019). When photosensitizers convert laser energy into heat, local tissue temperatures can rise to 45°C or higher, resulting in localized necrotic cell death (Hildebrandt et al., 2002). To effectively mediate PTT, nano-scale materials that absorb light across a wide range of the NIR spectrum and exhibit high photothermal conversion efficiency are critical. Suitable nanomaterials developed to date have included gold nanoparticles (NPs) (Zhang Y. et al., 2019; Alvi et al., 2021), carbon-based nanomaterials (Shen et al., 2020; Yu et al., 2020), and semiconductor nanostructures (Guo et al., 2017; Wang et al., 2019; Han et al., 2021). Fe_3_O_4_ NPs have previously been used selectively as contrast agents in the context of T2 magnetic resonance (MR) imaging, shortening the transverse relaxation time to improve negative contrast in T2-weighted images (Cheng et al., 2011; Cheng et al., 2012). These Fe_3_O_4_ NPs are highly stable, exhibit good photothermal conversion efficiency, and are both non-toxic and biocompatible under physiological conditions (Shen et al., 2015; Ren et al., 2016; Xiang et al., 2019; Lu et al., 2021). Polydopamine (PDA) is a biopolymer that exhibits good photothermal conversion efficiency and can be employed as a multi-functional coating agent (Beik et al., 2016), with PDA-coated nanomaterials having been employed for photothermal research and to diagnose and treat a variety of tumors (Xi et al., 2017; Schille et al., 2020).

In the present report, Fe_3_O_4_@PDA NPs were successfully synthesized and evaluated to establish their *in vitro* and *in vivo* utility as both contrast agents for T2 MR imaging and as therapeutic tools. Overall, our results clearly demonstrate that these Fe_3_O_4_@PDA NPs were able to effectively inhibit OS tumor growth and pulmonary metastasis, underscoring the value of leveraging these and similar nanomaterials for the diagnosis and treatment of OS.

## Materials and Methods

### Materials

Anhydrous ferric chloride (FeCl_3_), sodium acetate (NaOAc) and diethylene glycol (DEG) were purchased from Sinopharm Chemical Reagent Co., Ltd. (China). Dopamine hydrochloride (DA) was from Alfa Aesar (MA, United States). Sodium citrate was from Aladdin (Shanghai, China). All other chemicals were of analytical grade.

### Fe_3_O_4_ NP Preparation

After combining 20 ml of DEG and FeCl_3_ (324 mg, 2.0 mmol), 42.5 mg of and NaOAc (492 mg, 6.0 mmol) and 42.5 mg of sodium citrate (206 mg, 0.8 mmol) were added to this solution. The resultant mixture was placed in a Teflon-lined stainless-steel autoclave and heated to 210°C over 30 min, followed by a 10 h incubation at 210°C. The small magnetic Fe_3_O_4_ NPs produced through this reaction were then collected via centrifugation and sequentially rinsed using water and ethanol.

### Fe_3_O_4_@PDA NP Preparation

Fe_3_O_4_ NPs (14 mg) were suspended in 15 ml of Tri-Cl buffer (pH = 8.0, 0.1 M). The solution was then ultrasonicated for 5 min, after which DA (8.0 mg) was added and the mixture was constantly agitated for 12 h at 37°C. The resultant magnetic particles were then collected via centrifugation and rinsed using ethanol.

### Evaluation of Fe_3_O_4_@PDA NP Photothermal Properties

To explore the photothermal characteristics of the synthesized NPs, 1.0 ml of the Fe_3_O_4_@PDA NPs prepared at a range of concentrations (0, 20, 40, or 80 ppm) were irradiated for 12 min with a NIR laser (808 nm, 2.0 w/cm^2^). An online type thermocouple thermometer was then used to monitor the temperature of these NP solutions.

### *In Vitro* Magnetic Resonance Imaging

A range of NP concentrations was prepared in an aqueous solution containing 1% agar (0, 0.0625, 0.125, 0.25, 0.5, 1.0, 2.0 mM). T_2_-weighted MR imaging was conducted with a 9.4 T MRI magnet, with T_2_-weighted MR images and relaxation time T_2_ values being collected for analysis.

### *In Vivo* Magnetic Resonance Imaging

BALB/c nude mice (*n* = 5) received an intramedullary injection of 143B cells (1 × 10^7^) within the proximal tibia. Two weeks later, an orthotopic OS model had been established. When tumors had grown to 300 mm^3^, an Fe_3_O_4_@PDA solution was intravenously injected via the tail vein (5 mg/kg, 3.0 mg/ml in saline). Mice were assessed with a 3.0T MRI scanner in T_2_-weighted MR imaging mode at baseline and a 1, 2, 4, and 6 h post-injection, with T_2_-weighted imaging parameters being as follows: TR/TE = 3,000/50 ms, FOV = 60 mm, slice thickness = 1 mm, Image matrix = 256 × 256.

### Cell Culture and Treatment

For all *in vitro* experiments, 143B OS cells were cultured in DMEM supplemented with 10% FBS in a 37°C 5% CO_2_ incubator. To assess the cytotoxicity of NP preparations, these cells were plated in 96-well plates (5 × 10^3^/well) and cultured for 24 h, after which the supernatant was aspirated and cells were washed thrice with PBS. DMEM supplemented with a range of Fe_3_O_4_@PDA NP concentrations was then added for 24 h, after which an MTT assay was used to gauge cell viability. For appropriate wells, laser irradiation (808 nm, 2 W/cm^2^, 5 min) was performed prior to the MTT assay to gauge PTT efficacy.

In specific assays, 143B cells (1 × 10^4^/well) were separated into four treatment groups: control, saline+NIR, Fe_3_O_4_@PDA, and Fe_3_O_4_@PDA+NIR groups, with appropriate wells being cultured in the presence of 50 μg/ml of Fe_3_O_4_/PDA NPs. After treatment with or without NIR irradiation (808 nm, 1 W/cm^2^, 5 min), cells were stained for 20 min with Calcein-AM and propidium iodide (PI). Cells were then imaged via confocal microscopy. To evaluate apoptotic cell death, 143B cells were added to 6-well plates (3 × 10^5^/well) for 24 h, after which they were treated with appropriate NP solutions and were or were not subjected to NIR irradiation (808 nm, 1 W/cm^2^, 5 min). Cells were then harvested, rinsed thrice with PBS, stained with Annexin V-FITC/PI staining solution, and analyzed via flow cytometry. All assays were repeated three times, with three replicates per sample.

### Analysis of *In Vivo* PTT Efficacy

An orthotopic OS model was established in 20 BALB/c nude mice, as above. When tumors were 160–170 mm^3^ in size, these mice were randomized into four treatment groups (*n* = 5/group). Tumors in these mice were then injected with 50 μl of Fe_3_O_4_@PDA NPs (2 mg/ml) or 50 μl of 0.9% normal saline. Mice were then subjected to NIR laser irradiation (808 nm, 2 W/cm^2^, 8 min), with tumor temperature changes being monitored every minute with a NIR thermal imaging camera. Tumor weight and volume were measured every other day, with tumor volume being calculated as follows: V = ab^2^/2, where A and B respectively correspond to tumor length and width.

### Histological and Tissue Toxicity Analyses

After treatment for 3 weeks, a 1 ml blood sample was collected from each mouse following anesthetization, with alkaline phosphatase levels therein being measured. Mice were then euthanized, and tumors and major organs (brain, kidney, heart, liver, spleen, lungs) were collected and subjected to hematoxylin and eosin (H&E) staining. In addition, immunohistochemical (IHC) staining for CD31 and Ki-67 in the resultant tumor tissue sections was performed. All mouse studies were repeated three times, with three replicates per sample.

### Statistical Analysis

Data are given as means ± SD and were compared via one-way ANOVAs or independent sample t-tests using SPSS 19.0. An *α* = 0.05 test level was used, with *p* < 0.05 as the threshold of significance. *p < 0.05; **p < 0.01.

## Results and Discussion

**FIGURE 1:**
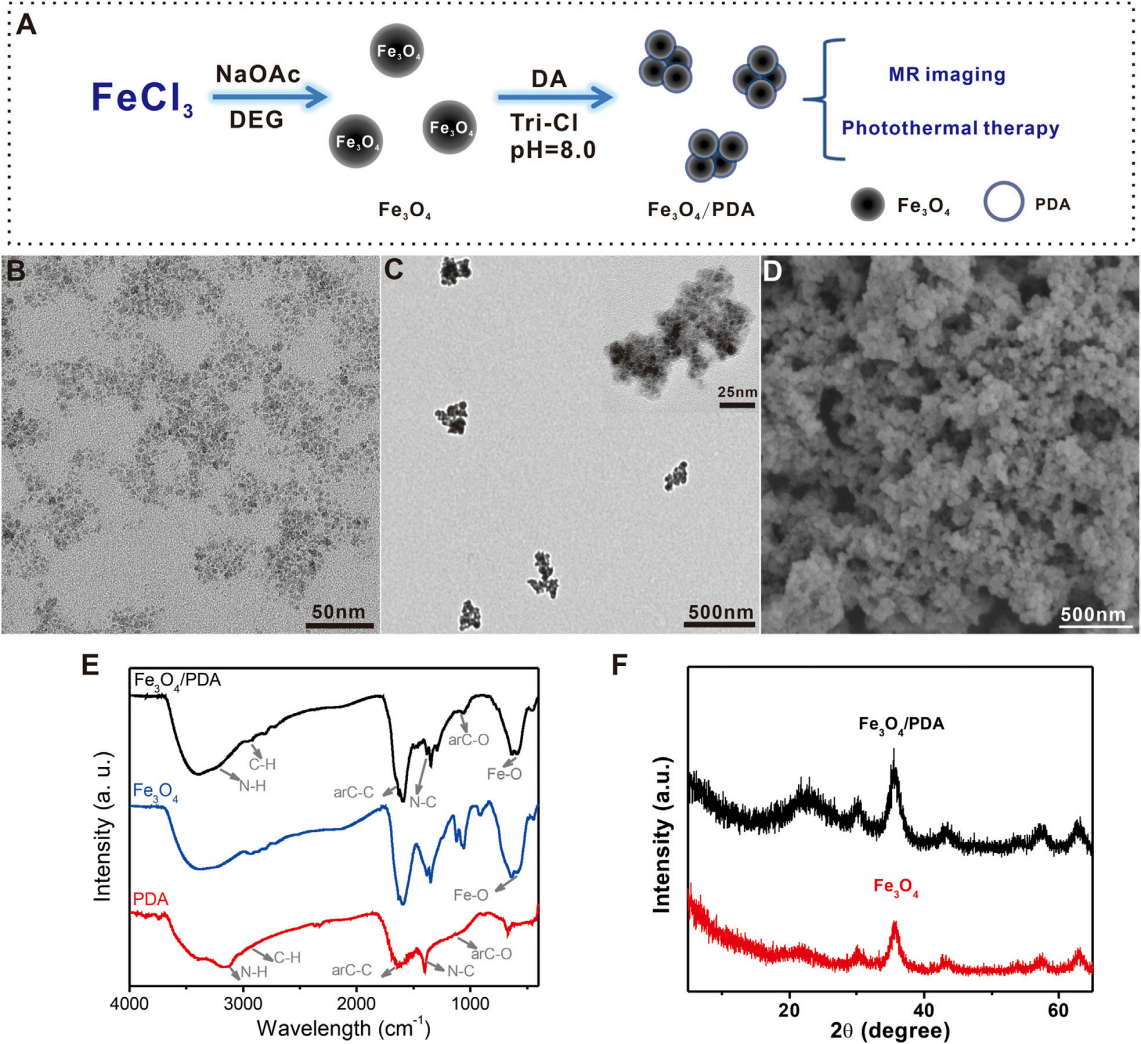
**(A)** Schematic overview of the Fe_3_O_4_@PDA nanocomposite preparation process. **(B,C)** TEM images of Fe_3_O_4_@PDA nanocomposites. Inset: High-resolution TEM images of Fe_3_O_4_@PDA nanocomposites. **(D)** SEM images of Fe_3_O_4_@PDA nanocomposites. **(E)** FTIR spectra for Fe_3_O_4_ NPs, PDA polymers, and Fe_3_O_4_@PDA nanocomposites. **(F)** Fe_3_O_4_ NP and Fe_3_O_4_/PDA nanocomposite XRD spectra.

Initially, Fe_3_O_4_ NPs were synthesized using ferric trichloride as precursor via a hydrothermal approach, with a PDA coating then being applied to yield Fe_3_O_4_@PDA nanocomposites (Figure 1A). When these NPs were evaluated via transmission electron microscopy (TEM) and scanning electron microscopy (SEM) (Figures 1B–D), they were found to be monodispersed spheres that were 3–9 nm in diameter. Following PDA coating, the size of these nanocomposites rose to 200–300 nm. To explore the structural characteristics of these Fe_3_O_4_@PDA nanocomposites, they were analyzed via high-resolution TEM, revealing small Fe_3_O_4_ NPs within the overall nanocomposite, consistent with successful Fe_3_O_4_ NP encapsulation within PDA polymers.

Next, the Fourier transform infrared (FTIR) spectra for PDA, Fe_3_O_4,_ and Fe_3_O_4_@PDA nanocomposites were generated (Figure 1E). PDA exhibited characteristic peaks at 3,210 cm^−1^ (ν_N–H_), 2,930 cm^−1^ (ν_C–H_) (Fang et al., 2010), 1,635 cm^−1^ (ν_arC-C_), 1,400 cm^−1^ (ν_N–C_), and 1,113 cm^−1^ (ν_arC-O_) (Liao et al., 2020) corresponding to N-H bond, C-H bond, aromatic ring, N-C bond, and C-O bond stretching vibrations, respectively. The Fe_3_O_4_ spectrum exhibited a characteristic peak at 584 cm^−1^(ν_Fe-O_) corresponding to the Fe-O bond. Fe_3_O_4_@PDA nanocomposites exhibited all characteristic peaks associated with both Fe_3_O_4_ and PDA polymers.

The crystalline structures of Fe_3_O_4_@PDA nanocomposites were assessed via X-ray diffraction (XRD) (Figure 1F). Peaks at (220), (311), (400), (422), (511), and (440) were clearly evident for both Fe_3_O_4_ and Fe_3_O_4_@PDA samples, consistent with the PDA polymer coating processing having not damaged the inverse spinel Fe_3_O_4_ (JCPDS NO. 19-0629)_._

**FIGURE 2:**
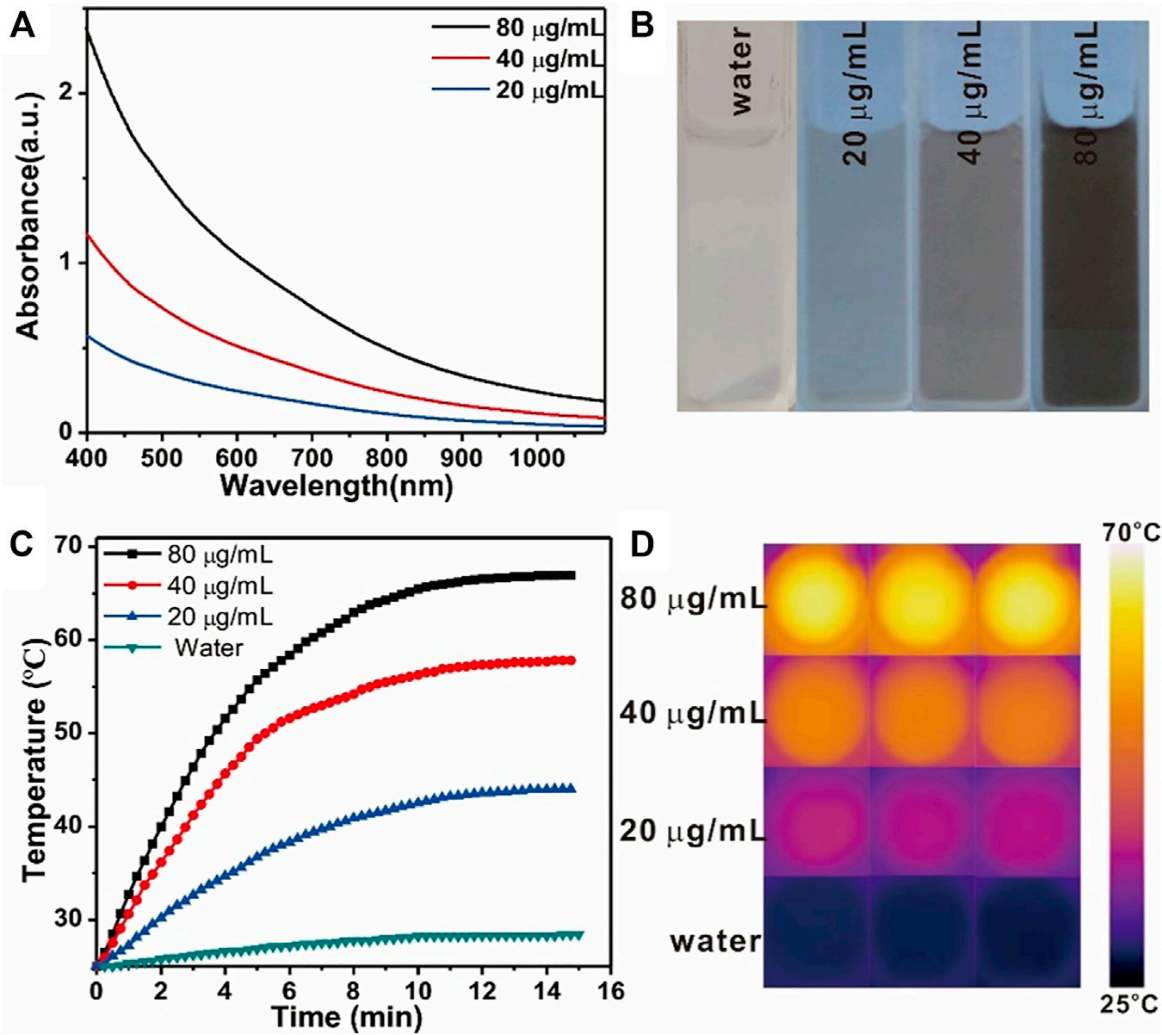
Vis-NIR spectra **(A)** and the images **(B)** of Fe_3_O_4_@PDA nanocomposites at a range of concentration levels. Temperature changes **(C)** and thermal images **(D)** for Fe_3_O_4_/PDA nanocomposites at a range of concentration levels over the course of NIR laser irradiation (808 nm, 0.8 W/cm^2^, 15 min).

The robust absorption of the prepared Fe_3_O_4_@PDA nanocomposites in the FTIR region (Figures 2A,B) led us to explore their photothermal efficacy. Upon NIR laser irradiation (808 nm, 1 W/cm^2^, 15 min), the temperature for a Fe_3_O_4_/PDA nanocomposite solution rose significantly up to 42°C in a dose-dependent manner as compared to pure water (17°C), underscoring the potential utility of these Fe_3_O_4_@PDA as photothermal agents. The photothermal conversion efficiency of Fe_3_O_4_@PDA nanocomposites was also calculated to be 31.9% (Supplementary Figure S1), which was slightly lower than the pure PDA nanomaterials.

**FIGURE 3:**
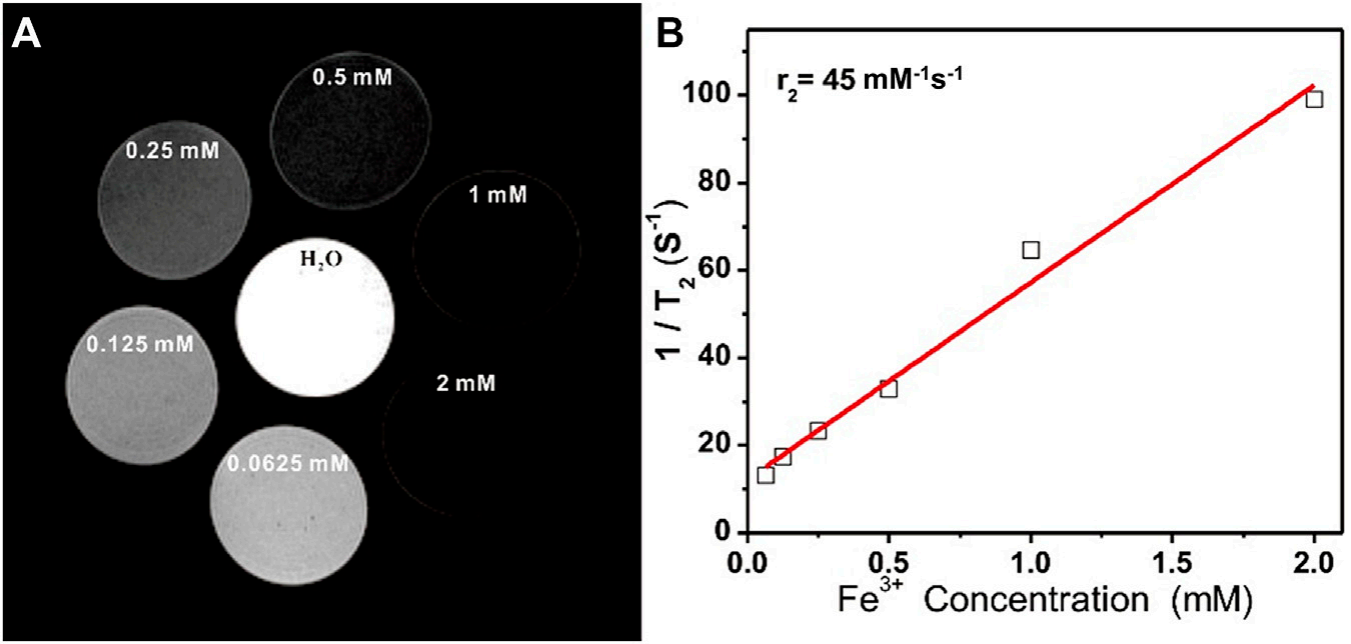
**(A)** T_2_-weighted MR images of Fe_3_O_4_@PDA nanocomposite solutions prepared at a range of Fe^3+^ concentrations. **(B)** T_2_ relaxation rates for Fe_3_O_4_@PDA nanocomposite solutions prepared at a range of Fe^3+^ concentrations.

T_2_-weighted MR images of prepared Fe_3_O_4_@PDA solutions were next generated using a 9.4 T MRI magnet, revealing that these nanocomposites mediated a clear dose-dependent contrast effect in the resultant images (Figure 3A), with a calculated T_2_ relaxivity (r_2_) of 45.0 mM^−1^ s^−1^ (Figure 3B).

An MTT assay was then performed to gauge the biocompatibility and toxicity of prepared NP solutions when applied to 143B cells and LO2 cells (Supplementary Figure S2). Overall, these Fe_3_O_4_@PDA nanocomposites exhibited low cytotoxicity, with 85.10% of cells remaining viable even at a nanocomposite concentration of 200 μg/ml. In order to test the stability of the Fe_3_O_4_@PDA nanpcomposites in cell culture medium, 200 ppm of the nanocomposites were dispersed in cell culture medium for 2 h no precipitation was observed, indicating that the Fe_3_O_4_@PDA nanpcomposites are very stable in culture (Supplementary Figure S3). Further MTT assay-based analyses of the PTT treatment efficacy of these nanocomposites were then performed, revealing a dose-dependent increase in cytotoxicity such that at a 50 μg/ml Fe_3_O_4_@PDA dose, 90.06% cell death was achieved following irradiation (808 nm, 2 W/cm^2^, 5 min), consistent with satisfactory *in vitro* PTT efficacy. When these nanocomposite concentrations were increased to 100 μg/ml, the increase in overall cell death was relatively limited (4.05%), and a dose of 50 μg/ml was thus selected for further experimentation.

**FIGURE 4:**
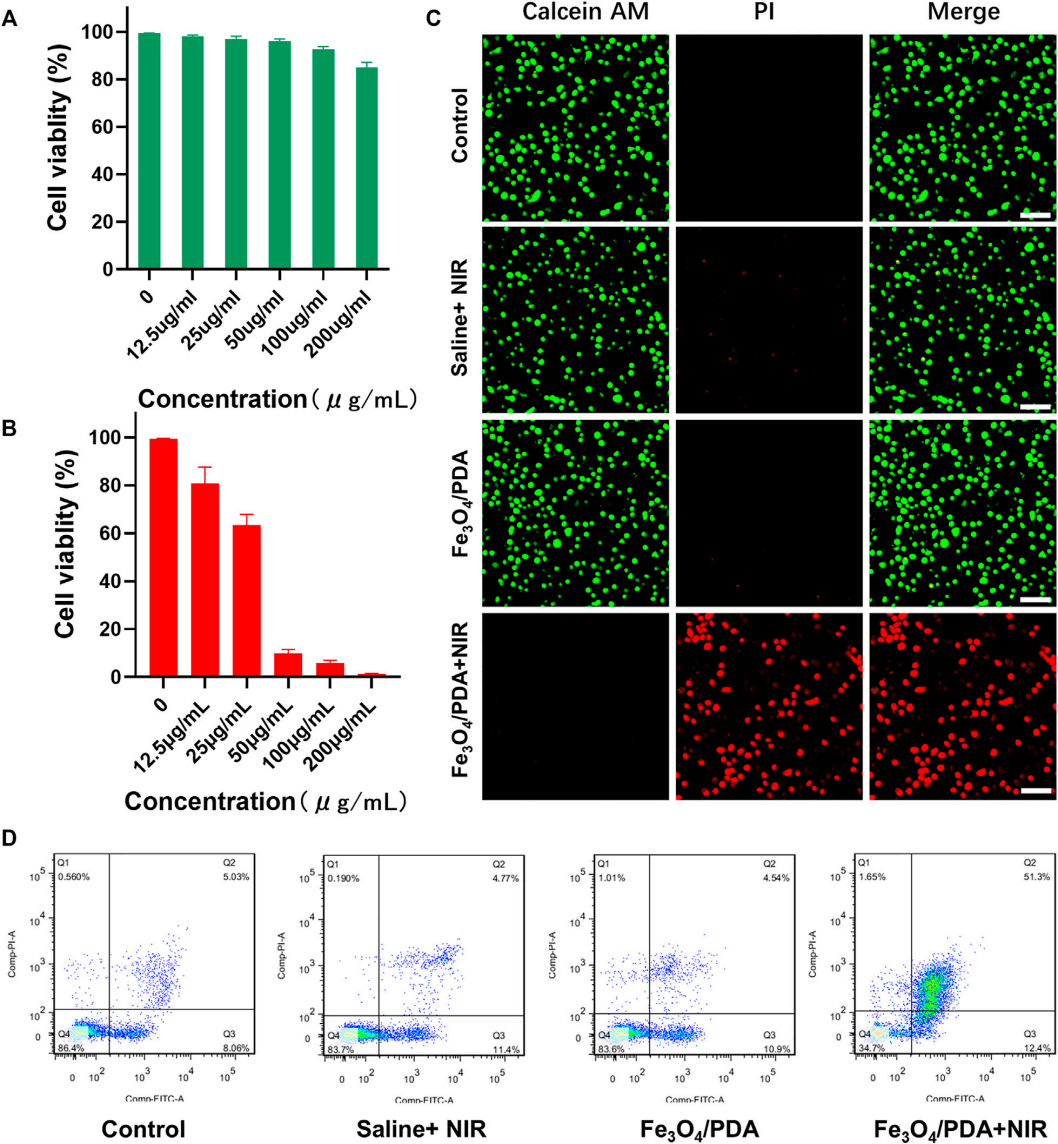
**(A)** The viability of cells treated with a range of Fe_3_O_4_@PDA concentrations for 24 h. **(B)** The viability of 143B cells following PTT, with irradiation being performed using an 808 nm laser in appropriate groups. **(C)** Fluorescent images of 143B cells in the indicated treatment groups that had undergone live/dead staining (Scale bar = 100 μm) **(D)** Representative flow cytometry plots for cells in the indicated treatment groups.

To more fully explore the effects of PTT treatment when using Fe_3_O_4_@PDA nanocomposites *in vitro*, Calcein-AM and PI were used to stain 143B cells in different treatment groups as a means of visualizing cell viability. While negligible cell death was evident in the first three treatment groups, near-total cell death was observed in the Fe_3_O_4_@PDA+NIR group (Figure 4C), confirming the ability of these nanocomposites to efficiently kill tumor cells upon laser-mediated excitation.

Following PTT, rates of apoptotic cell death in the control, saline+NIR, Fe_3_O_4_@PDA, and Fe_3_O_4_@PDA+NIR groups were 13.09, 16.17, 15.15, and 63.7%, respectively (Figure 4D), thus reaffirming the ability of these nanocomposites to mediate PTT.

**FIGURE 5:**
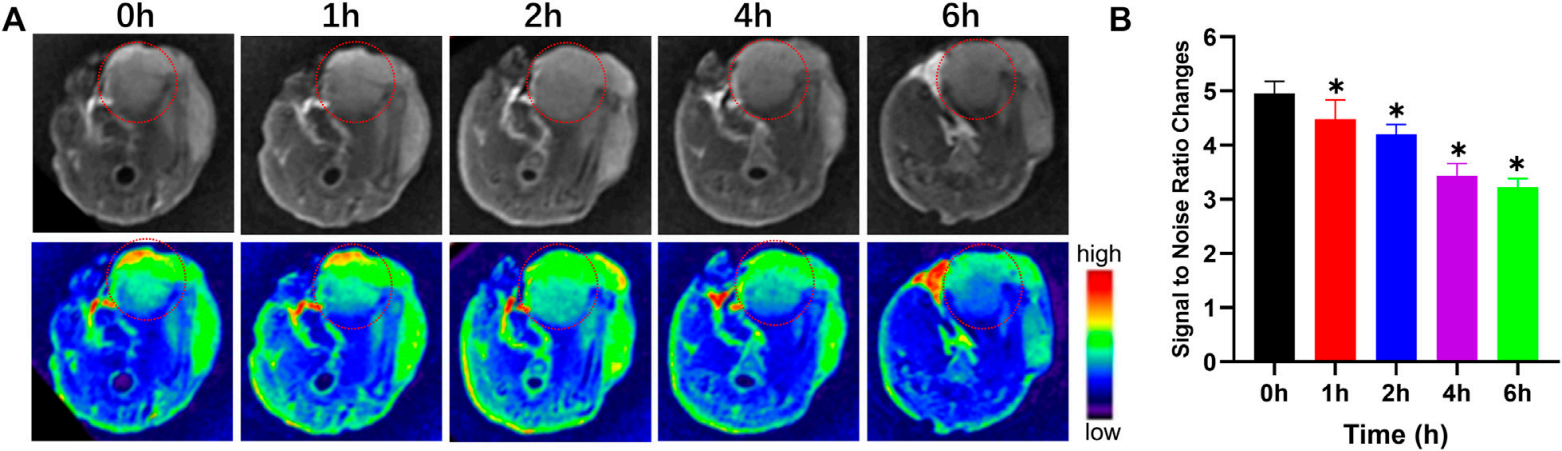
T2-weighted MR images **(A)** and changes in the signal-to-noise ratio **(B)** prior to and following the injection of a Fe_3_O_4_@PDA nanocomposite solution in orthotopic tumor-bearing mice.

To expand on the above results and explore the *in vivo* utility of our prepared nanocomposites, mice were intravenously injected with Fe_3_O_4_@PDA solutions via the tail vein (5 mg/kg of a 3.0 mg/ml solution in saline), after which T2-weighted MR images were captured with a 3.0 T instrument at baseline and at 1, 2, 4, and 6 h post-injection (Figure 5A). Prior to injection, the signal-to-noise ratio (SNR) for these orthotopic tumors was 4.92 ± 1.61, but it had risen to 3.23 ± 1.39 at 6 h post-injection, with the SNR for the tumor area being 34.34 ± 2.78% lower at this time point relative to baseline (Figure 5B). The contrast of these T2-weighted images gradually improved over time as evidenced by the darkening of the tumor area, thus improving overall MR imaging quality of these OS tumors in a manner that should be conducive to their early detection and treatment. This effect is likely primarily attributable to the enhanced permeability and retention (EPR) effect characteristic of the tumor-associated vasculature, which can enable iron oxide-based nanomaterials to remain in the tumor area for extended periods of time in a manner amenable to improved PTT treatment utilization.

**FIGURE 6:**
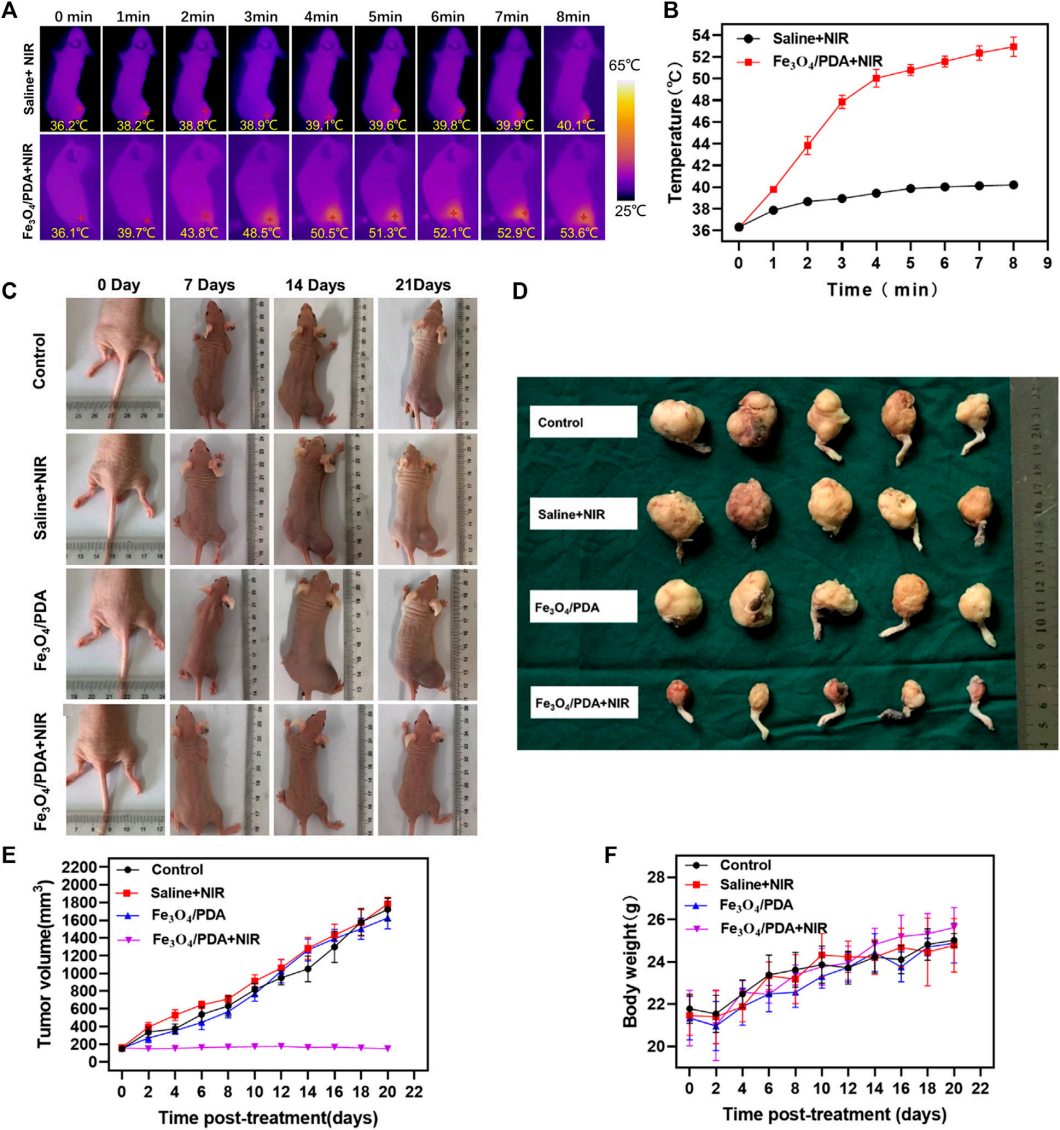
**(A)** Internal and external infrared thermal imaging results from mice at 1 h post-injection of Fe_3_O_4_@PDA or normal saline followed by NIR laser irradiation (808 nm, 2 W/cm^2^/, 8 min). **(B)** Changes in tumor temperature at the site of PTT were measured. **(C)** Images of orthotopic tumor-bearing mice at 1, 2, and 3 weeks post-PTT in the indicated treatment groups. **(D)** Images of resected tumors at 3 weeks post-treatment. **(E)** Changes in tumor volume over time. **(F)** Murine body weight values over time.

Next, orthotopic tumor-bearing nude mice were intratumorally injected with 50 μl of a 2 mg/ml Fe_3_O_4_@PDA solution or an equivalent volume of normal saline. Laser irradiation was then performed, with the temperature being monitored via infrared thermal imaging, revealing clear differences in temperature values between the saline+NIR and Fe_3_O_4_@PDA+NIR groups under laser irradiation (808 nm, 2 W/cm^2^, 5 min) (Figures 6A,B). Tumor temperatures rose to over 50°C within 4 min in the Fe_3_O_4_@PDA+NIR group, with local temperatures as high as 53.4 ± 0.3°C after 8 min.

These temperatures would be sufficient to induce thermal damage to the tumor, resulting in extensive necrotic cell death and consequent tumor ablation. In contrast, temperatures in the saline treatment group only rose to 40.0 ± 0.1°C.

In mice in the Fe_3_O_4_@PDA+NIR PTT treatment group, tumor growth was effectively controlled (Figures 6C,D). While tumor volumes in saline-treated control animals rose to 1,722.0 ± 112.6 mm^3^, they decreased to 146.0 ± 8.0 mm^3^ in animals that underwent Fe_3_O_4_@PDA+NIR treatment (Figure 6E). Tumor volumes for mice in the two other treatment groups were largely the same as those in control mice (*p* > 0.05). No significant differences in murine body weight were observed among groups over time (Figure 6F). In summary, these data indicated that Fe_3_O_4_@PDA+NIR treatment was sufficient to mediate the effective PTT-based ablation of orthotopic OS tumors in mice.

**FIGURE 7:**
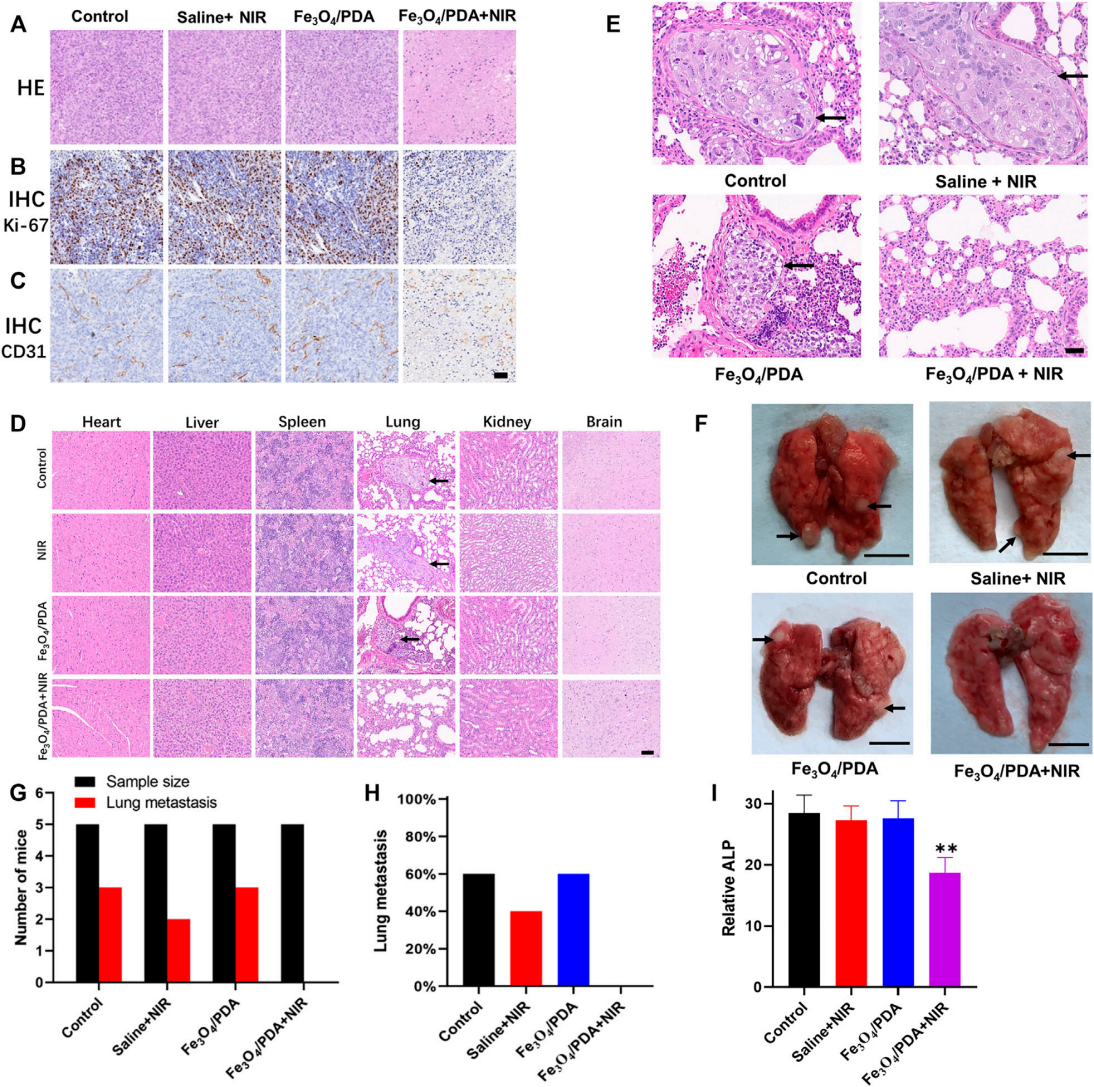
**(A)** Orthotopic tumors from mice in the indicated treatment groups were collected after 3 weeks and subjected to H&E staining (Scale bar: 100 μm). Tumors were additionally subjected to IHC staining for **(B)** Ki-67 and **(C)** CD31. **(D)** Major organs from mice in the indicated treatment groups were subjected to H&E staining, revealing lung metastases in the first three groups (Scale bar: 100 μm). **(E)** H&E staining of lung tissue sections from tumor-bearing mice revealed an absence of metastases in the Fe_3_O_4_@PDA+NIR group (Scale bar: 50 μm). **(F)** Pulmonary metastases (arrows) in the lungs of OS tumor-bearing mice (Scale bar: 5 mm) **(G)** Numbers of lung metastases in the indicated groups. **(H)** the proportion of mice exhibiting lung metastases in the indicated groups. **(I)** ALP values in the different groups.

Tumor tissue sections from mice in the different treatment groups were subjected to H&E staining, revealing no evidence of necrotic cell death in the control, saline+NIR, or Fe_3_O_4_@PDA groups, with cell morphology remaining intact (Figure 7A). In contrast, extensive necrotic tumor cell death and a loss of cellular morphology were evident in the Fe_3_O_4_@PDA+NIR group (Figure 7A). Ki-67 and CD31 immunohistochemical staining in the Fe_3_O_4_@PDA+NIR groups was reduced relative to that in the three other groups (Figures 7B,C).

Biosafety concerns are one of the primary barriers to the more widespread application of PTT. To that end, the histology of major organs collected from mice in the different treatment groups was assessed, revealing no evidence of necrotic cell death or morphological abnormalities following treatment in the brain, heart, spleen, kidneys, or liver (Figure 7D). Metastatic nodules were evident in the lungs of mice in all treatment groups other than the Fe_3_O_4_@PDA+NIR group (Figures 7D–F), while no metastases were observed in other organs. Metastatic tumor nodules in the lungs exhibited hyperstaining with heteromorphic changes and clear boundaries relative to the normal surrounding pulmonary tissue (Figures 7D,E). In contrast, lungs from mice in the three other treatment groups exhibited multiple solid metastatic nodules that were 1–3 mm in diameter with a fine texture (Figure 7F), with metastases being observed in 40–60% of mice in the first three treatment groups despite being evident in 0% of mice in the Fe_3_O_4_@PDA+NIR group.

Blood ALP levels were significantly lower for mice in the Fe_3_O_4_@PDA+NIR group, consistent with the ability of these nanocomposites to effectively inhibit OS tumor growth following NIR laser irradiation without inducing off-target toxicity in other major organs. Consistently, analyses of lung tissue samples from these mice indicated that Fe_3_O_4_@PDA+NIR treatment reduced both primary tumor size and the incidence of pulmonary metastasis, which has the potential to significantly improve OS patient prognostic outcomes (Lindsey et al., 2017; Gill and Gorlick, 2021). These results thus further underscore the promising utility of NP-based platforms for tumor-targeted PTT. However, additional pharmacokinetic and pharmacodynamic studies will be critical to the future clinical application of these materials.

## Conclusion

In conclusion, we herein developed Fe_3_O_4_@PDA nanocomposites that exhibit excellent photothermal properties and are well-suited to use in both MR imaging and PTT treatment applications. When intravenously administered to mice, these particles increased the tumor relaxation (R2) value significantly, thereby enhancing T2 imaging contrast and thus increasing the odds of successful early-stage OS tumor diagnosis. Further *in vitro* and *in vivo* analyses revealed that these Fe_3_O_4_@PDA nanocomposites were biocompatible and largely non-toxic. When excited via NIR laser irradiation, these Fe_3_O_4_@PDA nanocomposites mediated robust antitumor activity and prevented OS tumor pulmonary metastasis, underscoring the broad potential of these nanomaterials for use in the treatment of this deadly form of cancer.

## Data Availability

The original contributions presented in the study are included in the article/Supplementary Material, further inquiries can be directed to the corresponding authors.
