# Supplementary material for: Synthesis of Fe3O4/PDA Nanocomposites for Osteosarcoma Magnetic Resonance Imaging and Photothermal Therapy
Source: Front Bioeng Biotechnol. 2022 Mar 9;10:844540. doi: 10.3389/fbioe.2022.844540 (PMC8959548; doi:10.3389/fbioe.2022.844540)
Supplement: Supplementary file 1 [file DataSheet1.docx]

Supporting Information

Synthesis of Fe_3_O_4_/PDA nanocomposites for osteosarcoma magnetic resonance imaging and photothermal therapy

Yifei Zhang^1,2*^，Rende Ning^2^，Wei Wang ^2^, Yejin Zhou^2^，Yao Chen^1*^

^1^Department of Human Anatomy, West China School of Basic Medicine & Forensic Medicine, Sichuan University, Chengdu 610044, China

^2^Department of Orthopaedics, The Third Affiliated Hospital of Anhui Medical University, Hefei 230001, China

*** Correspondence:**Corresponding Author ：Yifei Zhang，[zyifei919@126.com](mailto:zyifei919@126.com)；Yao Chen，[chenyao62@scu.edu.cn](mailto:chenyao62@scu.edu.cn).


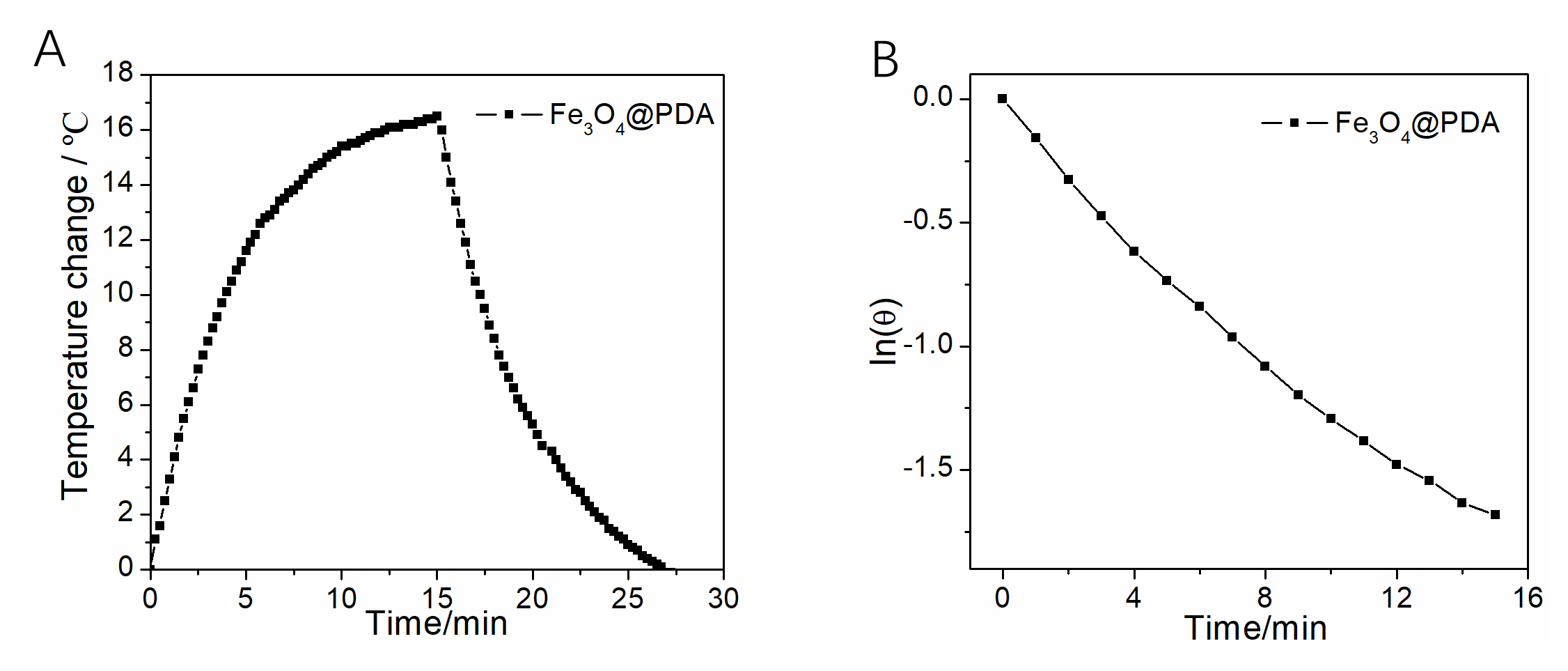


**Figure S1**. A) Photothermal effect of Fe_3_O_4_@PDA nanocomposites with NIR laser being irradiation for 15 min, and then the laser was shut off. B) Time constant for heat transfer from the system is determined to be **τ**_s_ = 8.9 s by applying the linear time data from the cooling period of (A). The photothermal conversion efficiency was calculated to be 31.9%.

**Figure S2**. Relative viability of LO2 cells incubated with Fe_3_O_4_@PDA nanocomposites at different concentrations.


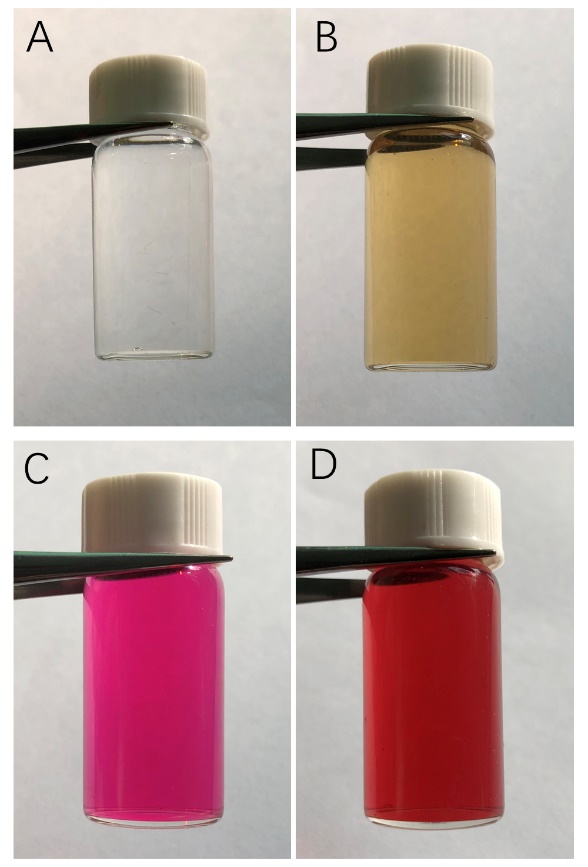


**Figure S3.** Photographs of PBS (A-B) and cell culture medium (DMEM) (C-D) in the absence or presence of nanocomposites. A-D) without Fe_3_O_4_@PDA nanocomposites, C-D) with Fe_3_O_4_@PDA nanocomposites at 200 ppm incubated for 2 h.


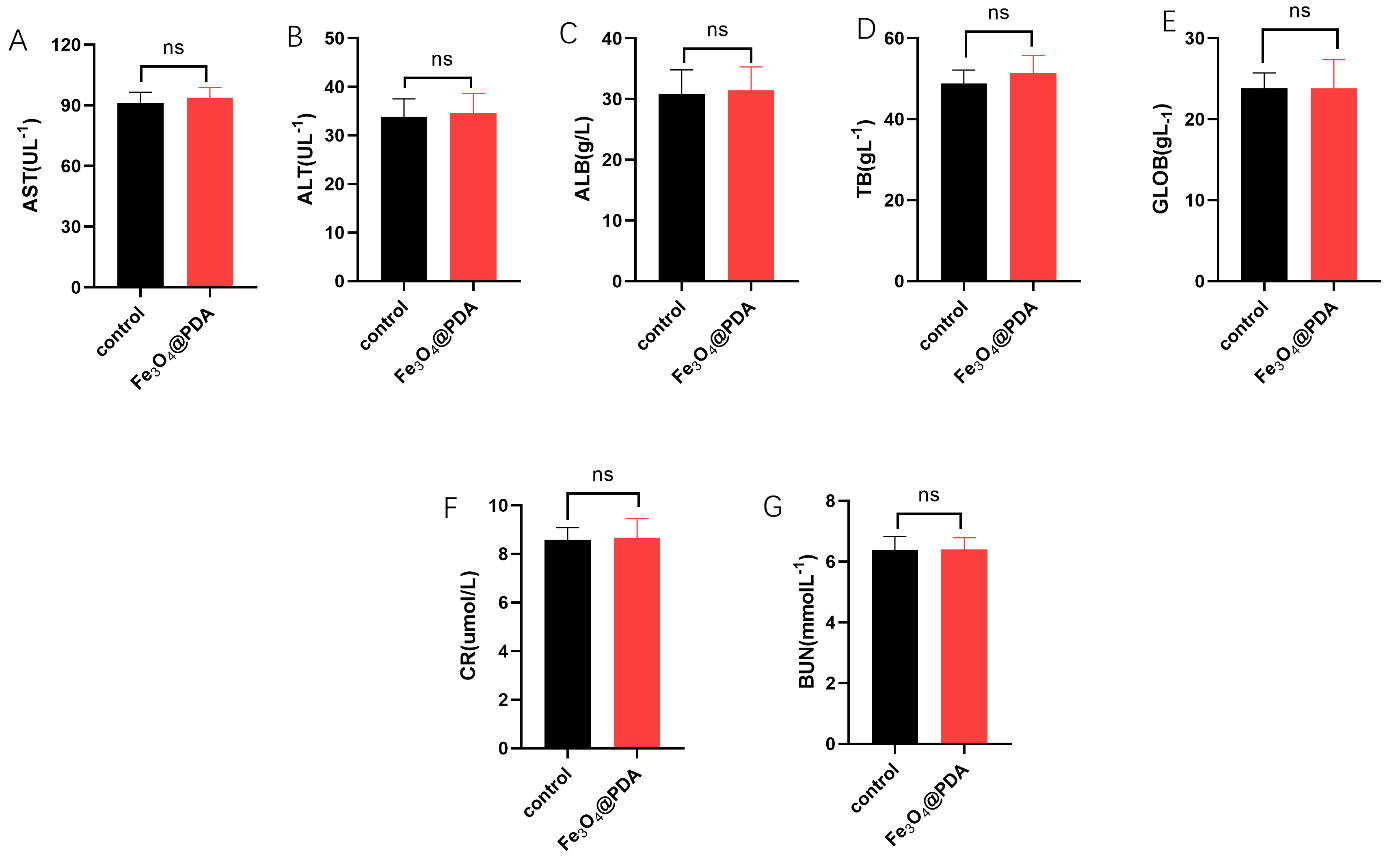


**Figure S4**. Blood biochemical analysis of mice 1 week after injection of Fe_3_O_4_@PDA.
